# Plasma cell‐free mitochondrial DNA declines in response to prolonged moderate aerobic exercise

**DOI:** 10.14814/phy2.12672

**Published:** 2016-01-12

**Authors:** Penny E. Shockett, Januka Khanal, Alina Sitaula, Christopher Oglesby, William A. Meachum, V. Daniel Castracane, Robert R. Kraemer

**Affiliations:** ^1^ Department of Biological SciencesSoutheastern Louisiana UniversityHammondLouisiana; ^2^ Department of Obstetrics and GynecologyTexas Tech University Health Sciences Ctr.OdessaTexas; ^3^ Department of Kinesiology and Health StudiesSoutheastern Louisiana UniversityHammondLouisiana

**Keywords:** Cell‐free DNA, cell‐free mitochondrial DNA, damage‐associated molecular pattern, DAMP, exercise, inflammation

## Abstract

Increased plasma cell‐free mitochondrial DNA (cf‐mDNA), a damage‐associated molecular pattern (DAMP) produced by cellular injury, contributes to neutrophil activation/inflammation in trauma patients and arises in cancer and autoimmunity. To further understand relationships between cf‐mDNA released by tissue injury, inflammation, and health benefits of exercise, we examined cf‐mDNA response to prolonged moderate aerobic exercise. Seven healthy moderately trained young men (age = 22.4 ± 1.2) completed a treadmill exercise trial for 90 min at 60% VO_2_ max and a resting control trial. Blood was sampled immediately prior to exercise (0 min = baseline), during (+18, +54 min), immediately after (+90 min), and after recovery (R40). Plasma was analyzed for cf‐mDNA, IL‐6, and lactate. A significant difference in cf‐mDNA response was observed between exercise and control trials, with cf‐mDNA levels reduced during exercise at +54 and +90 (with or without plasma volume shift correction). Declines in cf‐mDNA were accompanied by increased lactate and followed by an increase in IL‐6, suggesting a temporal association with muscle stress and inflammatory processes. Our novel finding of cf‐mDNA decline with prolonged moderate treadmill exercise provides evidence for increased clearance from or reduced release of cf‐mDNA into the blood with prolonged exercise. These studies contrast with previous investigations involving exhaustive short‐term treadmill exercise, in which no change in cf‐mDNA levels were reported, and contribute to our understanding of differences between exercise‐ and trauma‐induced inflammation. We propose that transient declines in cf‐mDNA may induce health benefits, by reducing systemic inflammation.

## Introduction

The immune response is critical for clearance of microbial pathogens but also contributes to many diseases involving inflammation (Maury and Brichard 2010; Davis et al. 2011; Cappellano et al. 2013). While exercise is prescribed for disease treatment and prevention, the stress produced by exercise also promotes inflammation (Calle and Fernandez 2010; Walsh et al. 2011; Raschke and Eckel 2013). Several recent studies have suggested that health benefits result when inflammation such as that stimulated by exercise promotes dampened systemic inflammation (Gleeson et al. 2011; Lancaster and Febbraio 2014).

Interestingly, like inflammation‐inducing molecules from microbes (i.e., pathogen‐associated molecular patterns, PAMPs), certain cellular molecules (damage‐associated molecular patterns, DAMPs) released by diseased, injured, or dying cells as “stress” or “damage/danger” signals also promote inflammation (Shen et al. 2013). These “sterile” mediators of inflammation, like microbial components, bind pattern recognition receptors (PRRs) on inflammatory immune cells triggering release of cytokines and other molecules and inflammatory responses including production, migration, and activation/phagocytic activity of neutrophils.

Of interest here, cell‐free mitochondrial DNA (cf‐mDNA), a DAMP and sterile mediator of inflammation was recently shown to be elevated in the blood of injury‐induced trauma patients, where it appears to promote systemic inflammatory response syndrome (SIRS) (Zhang et al. 2010). Mitochondria likely evolved by endosymbiosis involving aerobic bacteria engulfed by an ancestral anaerobic cell. Thus, mDNA shares many similarities with bacterial DNA, including size, circularity, lack of histones, low CpG methylation, and ability to activate inflammatory cells via PRRs (reviewed in [Nakahira et al. 2015]). Trauma studies revealed that inflammation, neutrophil activation, and organ injury can be mediated by cf‐mDNA binding to Toll‐like receptor‐9 (TLR‐9, also known to bind bacterial DNA) on neutrophils (Zhang et al. 2010).

Exercise, like trauma, is a form of bodily stress that promotes oxidative damage, cytokine secretion, and inflammation that can lead to SIRS (Fehrenbach and Schneider 2006). Even moderate exercise, if unaccustomed or prolonged, can result in muscle damage (Kraemer and Brown 1986) that subsides as muscle cells experience recovery and repair (Paulsen et al. 2012; Pillon et al. 2013; Raschke and Eckel 2013). Cytokine/myokines, such as Il‐6, released from hematopoietic cells during trauma and secreted by muscle cells responding to heavy exercise, participate in “cross‐talk” between exercising muscle and inflammatory cells and are secreted into the blood where they may influence systemic inflammatory responses (Fehrenbach and Schneider 2006; Pedersen and Febbraio 2012; Pillon et al. 2013; Raschke and Eckel 2013). A “compensatory anti‐inflammatory immune response (CARS)” to SIRS (Bone 1996) involves shifting cytokine profiles (e.g., IL‐6), which likely plays a major role. Current concepts regarding the health benefits of exercise incorporate the ability of exercise to stimulate and resolve inflammation promoting tissue repair.

While cf‐mDNA is associated with inflammation and SIRS during trauma, altered levels of cf‐mDNA are also associated with endothelial disfunction in development of diabetes mellitus, sepsis, cardiac disease, and atherosclerosis, with increased cf‐DNA levels detected in individuals with cancer and autoimmune diseases (Alvarado‐Vasquez 2015). After exercise, although plasma cell‐free nuclear DNA (cf‐nDNA) levels were shown to transiently increase after both heavy acute treadmill and cycling exercise (Fatouros et al. 2010; Beiter et al. 2011, 2014), and in proportion to training load and acute phase response during chronic excessive resistance exercise (Fatouros et al. 2006)**,** cf‐mDNA, a known inflammatory mediator, did not appear to change with short‐term, intense exercise.

Cellular source(s) of cf‐DNA have been controversial. Recently, exhaustive short‐term treadmill exercise experiments revealed concomitant increases in plasma nuclear DNA (cf‐nDNA), deoxyribonuclease I (DNAse I), and disintegrating neutrophils with extruded chromatin structures, consistent with the presence of microbe‐capturing neutrophil extracellular traps (NETs) (Beiter et al. 2011, 2014, 2015). No increase in cf‐mDNA was observed. In one study, NETs were reported to be induced by acute severe cycling exercise only in sedentary subjects (Syu et al. 2013). In another study, 60 min of moderately intense cycling led to increases in plasma cf‐nDNA and DNAse I, regardless of exercise training status (Beiter et al. 2014). Based on expression of neutrophil, inflammatory, and cytokine markers compared with markers of muscle damage, it was concluded that early increase in cf‐nDNA and DNAse I post‐strenuous acute exercise did not result from muscle damage, but rather from NETs (Beiter et al. 2014). Other studies also reported a transient rise in cf‐nDNA (but not cf‐mDNA) immediately after and a more gradual increase in markers of muscle damage (and inflammatory acute phase response) still rising 24 h post acute exhaustive treadmill exercise (Fatouros et al. 2010; Beiter et al. 2011; Breitbach et al. 2012). It has been proposed that NET extrusion and resolution (involving DNAse I) under certain “safe” endurance exercise conditions free from excessive stress/training loads, or insufficient recovery, may contribute to maintenance of immune homeostasis and protection from chronic inflammation (Beiter et al. 2015).

Current models propose NETosis (NETs), exocytosis, and cell‐surface release as acute short‐term intense exercise sources of rapidly released cf‐DNA and apoptosis (of damaged muscle cells or postinflammatory lymphocytes) as chronic/long‐term exercise‐induced sources of slowly accumulating cf‐DNA (Breitbach et al. 2012). In trauma, cf‐mDNA is thought to derive primarily from injured tissue (Zhang et al. 2010; Itagaki et al. 2015), and one might anticipate a similar response from exercising muscles.

Thus, we conducted a study to determine the effects of 90 min of moderate prolonged aerobic exercise on plasma concentrations of cf‐mDNA. Our hypothesis was that a longer bout of exercise would increase plasma levels of cf‐mDNA, as had been observed generally for cf‐DNA in other exercise studies (Breitbach et al. 2012) and specifically for cf‐mDNA in trauma patients (Zhang et al. 2010). However, our results suggest that prolonged moderate treadmill exercise can transiently reduce plasma cf‐mDNA levels, while inducing a rise in plasma IL‐6. We discuss these results in the context of contrasting studies that found no change in cf‐mDNA with exercise and of the expanding understanding of cf‐mDNA as a biomarker of inflammation in exercise, trauma, and disease. Additionally, we propose a model in which transient declines in cf‐mDNA with certain exercise regimens may contribute to exercise‐induced health benefits resulting from reduced systemic inflammation.

## Materials and Methods

### Prolonged moderate aerobic exercise trials

#### Human subjects

This study was conducted in accordance with and approved by the Southeastern Louisiana University IRB. Seven young male subjects were prescreened, signed informed consent to participate in the study, and completed a 3‐day food record as well as a medical history questionnaire. Subjects were a subset of a previous study investigating glucoregulatory hormone responses to exercise (Kraemer et al. 2011) and met the following criteria for participation: (1) between ages 18 and 35 years; (2) not taking any prescription medications, (3) not adhering to a diet that would affect metabolic responses to exercise, and (4) had no history of metabolic or cardiorespiratory disease.

#### Preliminary trials

All seven subjects reported to the laboratory and completed a preliminary exercise trial to determine body composition and maximal oxygen uptake (VO_2_ max). Body composition was first determined using a 7‐site skinfold assessment (Kraemer et al. 2011). The subjects then completed a graded exercise treadmill test (Kraemer Protocol, [Kraemer et al. 2011]) beginning at 2.5 miles·h^−1^, 4% grade, and progressing by 1 mile·h^−1^ every 2 min until exhaustion. Expired gas volumes, F_E_O_2_, and F_E_CO_2_ were continually collected and analyzed using a metabolic cart (ParvoMedics 2400, Sandy, UT). VO_2_max was reached when either of the following was met: the primary criterion of a plateau in VO_2_ with increased workload or two of three secondary criteria: (1) reaching predicted maximal heart rate; (2) respiratory exchange ratio >1.1; or (3) rating of perceived exertion (RPE, 15‐point Borg scale) of 19 or 20. Descriptive data are shown in Table 1.

**Table 1:** Descriptive data

Measure	Mean (±SD)
Age (year)	22.43 (1.15)
Height (cm)	178.36 (6.1)
Weight (kg)	73.56 (8.1)
Body mass index (kg/m^2^)	23.25 (3.63)
Fat (%)	11.92 (6.31)
VO_2_ max (mL/kg/min)	57.33 (8.49)

#### Exercise and control trials

Before the exercise and control trials, the subjects (who were the same for exercise and control trials) were instructed to (1) abstain from exercising and drinking alcohol 48 h before the trial; (2) maintain their normal diet; (3) fast from at least midnight the night before; and (4) report to the laboratory at 8:00 am after consuming a 350‐kcal liquid meal (Ensure Plus^™^) at 7:00 am to standardize pretrial meal consumption. The calculated kcal expenditure for the 90‐min exercise trial was 700–900 kcal. For the exercise trial, an intravenous catheter was inserted at 8:30 am the morning of the trial. Blood patency was maintained using a saline lock. In the present study, blood samples were collected and subsequently analyzed at the following time points across the exercise and control trials. A resting blood sample (28 mL) was drawn at 9:30 am (0 min = baseline), at which time subjects began exercising on the treadmill. VO_2_ was continually monitored during 90 min of exercise, and treadmill speed and grade were adjusted to maintain a VO_2_ of 60% of the previously determined VO_2_ max. Blood samples were collected during (+18, +54 min), immediately after (+90 min), and after recovery from exercise R40 for cf‐mDNA or R60 for IL‐6. For the control trial, the blood sampling protocol time points were identical to the exercise trial; however, the same subjects rested in a seated position with no exercise or measurement of VO_2_. Exercise and control trials were counterbalanced, with at least 1 month between trials.

#### Blood sample processing

Blood samples for cf‐mDNA and IL‐6 analysis were collected in chilled EDTA tubes. Blood samples for lactate analysis were collected into sodium oxalate/potassium fluoride tubes. Blood cells were removed from plasma by centrifugation for 5 min at 800 g and plasma was frozen at −80°C. For cf‐mDNA isolation, plasma was further centrifuged for 5–7 min at 2600 g to remove platelets.

#### Isolation and analysis of plasma cf‐mDNA

cf‐mDNA from 200 *μ*L plasma was isolated in triplicate (for most samples) using a Qiamp DNA Mini DNA extraction kit (Qiagen, Valencia, CA) according to the manufacturer's instructions, with elution into a 200 *μ*L volume. Initial pilot polymerase chain reaction (PCR) amplifications suggested that mDNA was detected using several human mDNA gene primer pairs previously described (Zhang et al. 2010), while nuclear DNA was not detected using primers for human RAG2 (FOR, RAG2R GCACAGTCTTGCCAGGAGGAATC; REV, hRAG2REV TCTTTGGGGAGTGTGTAGAGC). Additionally, no bacterial 16s rDNA was detected (Zhang et al. 2010); 1 *μ*L of a 1:15 dilution of DNA was amplified using 30 cycles of PCR (55°C annealing) with primers designed to detect human mitochondrial CytC oxidase subunit III (Zhang et al. 2010). Reactions were run on 1.5% agarose gels stained with EtBr, imaged on a ChemiDoc Gel Imager (BioRAD, Hercules, CA), and quantitated using Image Lab image analysis software (BioRAD, Hercules, CA). Dilution of DNA prior to analysis allowed amplification and detection within the linear range of the PCR assay and imager detection sensitivity.

#### Measurement of plasma IL‐6, lactate, hematocrit, and hemoglobin

IL‐6 was measured in (0, +54, +90, and R60) plasma samples using an EIA kit (Caymen Chemical, Ann Arbor, MI) according to the manufacturer's instructions. IL‐6 results derive from six subjects, because for one subject, plasma samples were unavailable. Plasma lactate concentrations were determined for all subjects and time points using a commercial enzymatic spectrophotometric reagent (Pointe Scientific, Canton, OH). Hemoglobin concentrations from whole blood were determined using an enzymatic colorimetric method (Pointe Scientific), and hematocrit was determined using a microhematocrit method. Hemoglobin and hematocrit were used to determine plasma volume shifts over time (Kraemer et al. 2011).

### Data analysis

Means reported for cf‐mDNA were derived from averages of % baseline, in most cases, from triplicate DNA isolations from each plasma sample. Statistical analyses were performed using SPSS PASW Statistics 18 (IBM Corp, Somers, NY) and Excel. Cf‐mDNA, IL‐6, and lactate data were analyzed using a trial × time repeated‐measures ANOVA. If significant effects were indicated by ANOVA (alpha level, 0.05), post hoc comparisons of control and exercise trials at specific time points or between time points within a trial were made using paired two‐tailed Student's *t*‐tests (alpha level, 0.05). Shapiro–Wilk normality tests indicated that data sets were not inconsistent with a normal Gaussian distribution.

## Results

### Transient decline in plasma cf‐mDNA with 54‐ and 90‐min prolonged moderate aerobic treadmill exercise

**Figure 1:**
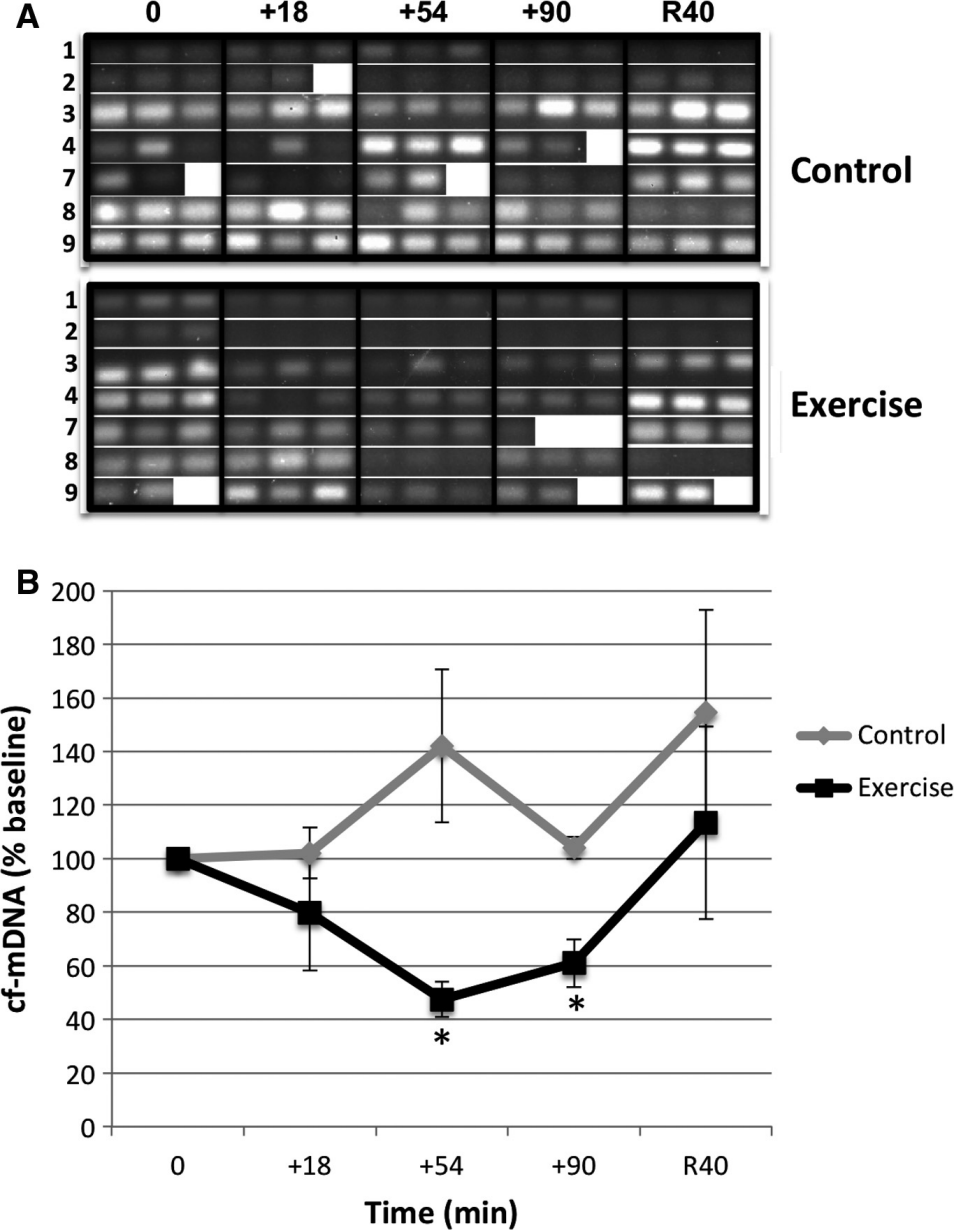
Plasma cf‐mDNA levels decline at +54 and +90 during prolonged moderate treadmill exercise as measured by PCR for CytC oxidase subunit III: (A) PCR products as observed on 1.5% agarose gels and (B) quantitation of results from gels. *Significant difference (*P* < 0.05) between trial time points. Values represent PVS‐corrected mean ± SEM (*N* = 7, same subjects in exercise and control trials, subject number listed in rightmost column in A). Note that results were similar with no PVS correction. Values represent means of seven subjects derived from averages (of individual subjects % baseline values), in most cases from triplicate DNA isolations from available plasma samples.

Amplification of CytC oxidase subunit III and analysis of percent change across time by 2 (trial) × 5 (time) ANOVA indicated a significant trial effect between exercise and control trials [*F*(1,12) = 5.73, *P *=* *0.034] for plasma cf‐mDNA levels (Fig. 1). Levels of cf‐mDNA in the exercise samples differed significantly from those of the control trial at +54 (*P *=* *0.01) and +90 (*P *=* *0.012) of exercise, with concentrations of cf‐mDNA reduced at +54 (47.5% baseline) and +90 (61.02% baseline) of exercise. Although reported with plasma volume shift (PVS) correction, the correction (of shifts mostly ranging from −13 to −1 at +54 and +90) only minimally influenced the results. This indicated that both the effective levels of plasma cf‐mDNA and the occurrence and/or clearance of cf‐mDNA were altered during the exercise trial.

### Transient increase in plasma IL‐6 with 90‐min prolonged moderate aerobic treadmill exercise

To determine whether exercise impacted inflammatory processes in these trials that might be related to the decline in cf‐mDNA observed, we measured IL‐6 levels by ELISA in plasma collected during the trials.

**Figure 2:**
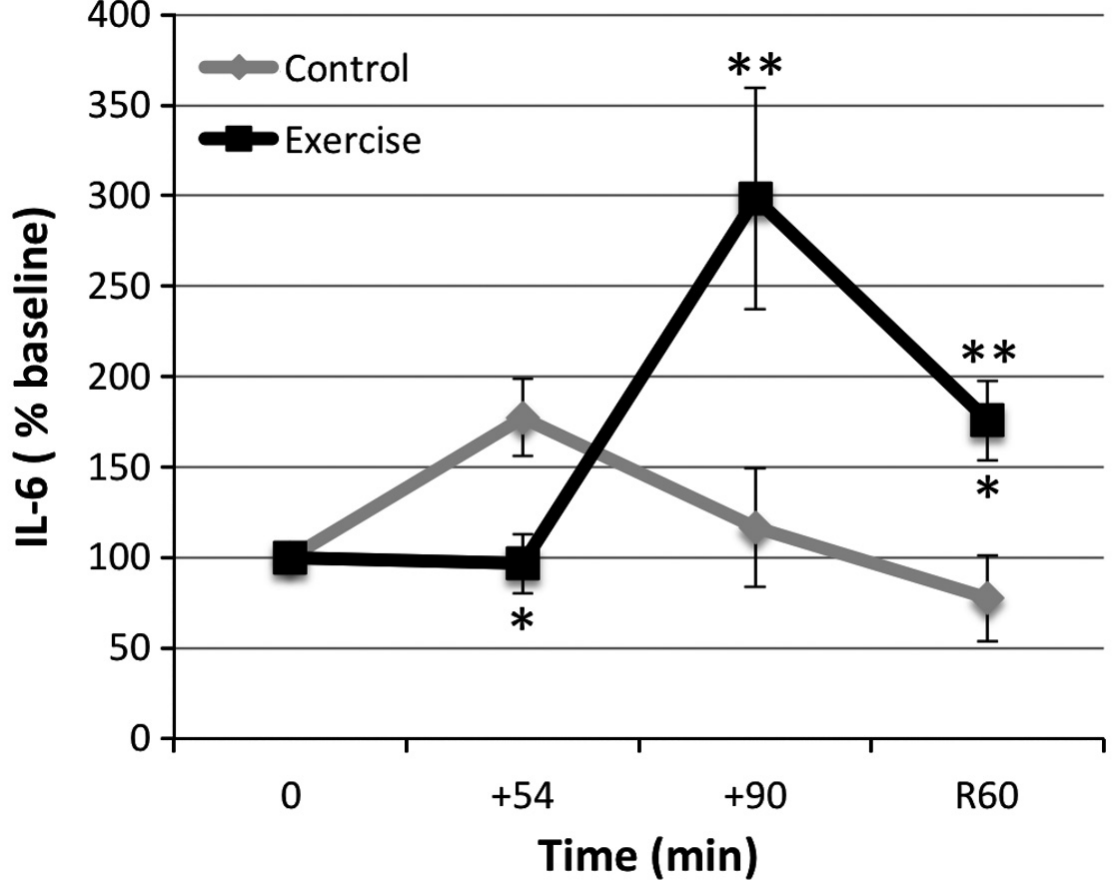
Plasma IL‐6 levels increase at +90 during prolonged moderate treadmill exercise as measured by ELISA: *Significant difference (*P* < 0.05) between trial time points and **between exercise time point and baseline (0 min). Values represent PVS‐corrected mean ± SEM (*N* = 6, same subjects in exercise and control trials). Note that results were similar with no PVS correction, and that in two instances, missing control data points (S2, +54, and S3, +90) were replaced with mean values at the time point.

Using a 2 (trial) × 4 (time) ANOVA for plasma IL‐6 response, significant time [*F*(1.5, 14.6) = 6.11, *P *=* *0.018], trial × time interaction [*F*(1.5, 14.6) = 9.52, *P *=* *0.004], and trial [*F*(1, 10) = 5.09, *P *=* *0.048] effects were observed (Fig. 2). With exercise, IL‐6 concentrations were significantly elevated at +90 (298.3% baseline, *P *=* *0.023) and R60 (175.9% baseline, *P *=* *0.019) (Fig. 2). These data suggested that the initial decline in cf‐mDNA seen with exercise was followed by a subsequent increase in IL‐6 by the end of the trial, which was still evident after an hour of recovery. Thus, the decline in cf‐mDNA observed may be associated with inducing inflammatory processes.

### Sustained increase in lactate with 18‐ to 90‐min prolonged moderate aerobic exercise

**Figure 3:**
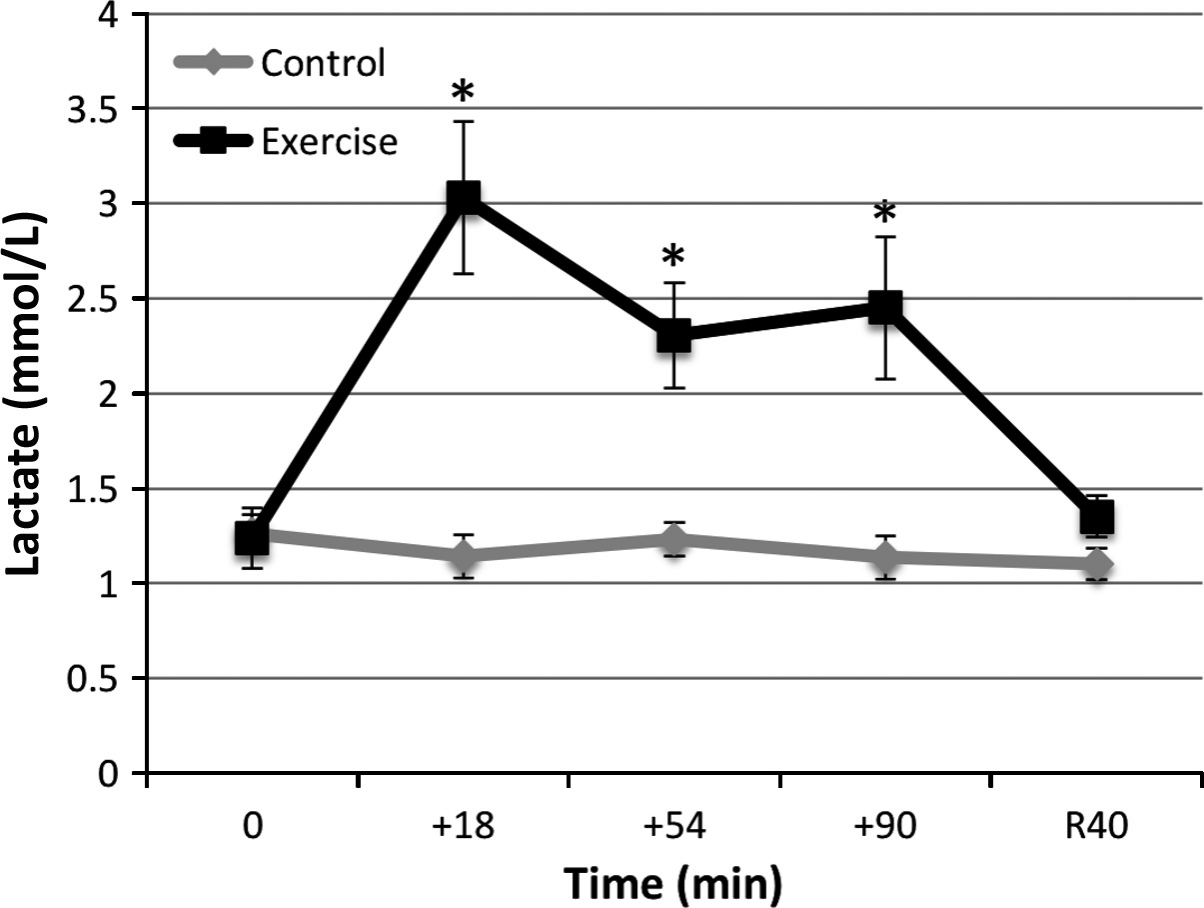
Plasma lactate levels are increased over the course of the prolonged moderate treadmill exercise trial. *Significant difference (*P* < 0.05) between trial time points and between exercise time point and baseline (0 min). Values represent mean ± SEM (*N* = 7, same subjects in exercise and control trials) and were not corrected for PVS.

Increased lactate concentration served as an additional indicator of exercise exertion and muscle stress in the trials (Fig. 3). A 2 (trial) × 5 (time) ANOVA indicated significant time [F(2.33, 28) = 9.73, *P *=* *0.000], trial × time [F(2.33, 28) = 10.6, *P *=* *0.000], and trial [*F*(1, 12) = 17.08, *P *=* *0.001] effects. A significant difference between exercise and control trials was seen by 18 min (*P *<* *0.004) and was maintained at 54 (*P *<* *0.01) and 90 (*P *<* *0.01) min of exercise. A significant difference was also observed between baseline lactate values and those at +18, +54, and +90 min of exercise (*P *=* *0.006, *P *<* *0.02, and *P *<* *0.03, respectively). Maximal lactate increase (2.4‐fold, to 3 mmol/L) was observed at +18 and was maintained at 2.3 (+54) and 2.45 (+90) mmol/L during the exercise trial.

## Discussion

This is the first study to show that prolonged moderate aerobic exercise can result in decline of cf‐mDNA. Most existing studies of exercise‐induced changes in circulating cf‐DNA levels have reported increases post exercise, but do not specifically examine cf‐mDNA. The subtitle of a recent review on the subject suggests that cf‐DNA may be “An Up‐Coming Molecular Marker in Exercise Physiology” (Breitbach et al. 2012). Recent studies specifically examining cf‐mDNA of trauma patients showed elevated plasma levels, which appeared to promote inflammation involving neutrophil activation (Zhang et al. 2010). For these reasons, our expectation was to observe increased plasma cf‐mDNA levels with prolonged moderate aerobic exercise. Unexpectedly, we found that prolonged moderate aerobic treadmill exercise led to a decline in plasma cf‐mDNA, which was followed by a transient increase in plasma IL‐6. While the basis of the reduction in cf‐mDNA levels remains unknown, several nonmutually exclusive possibilities should be considered.

First, mDNA, a DAMP, is known to bind endosomal TLRs located in inflammatory cells, such as neutrophils and monocytes. Recent transcriptome analysis in neutrophils of human subjects completing 1 h endurance cycling plus 1 h running demonstrated early increases at 3 h of plasma indexes of muscle damage, neutrophils, IL‐6, Il‐10, and IL‐1 receptor and TLRs, post exercise (Neubauer et al. 2013). Negative regulators of immunity were also upregulated, and neutrophils were back to resting by 48 h post exercise indicative of a compensatory response (Neubauer et al. 2013). Upregulation/downregulation of neutrophil genes involved in inflammation and innate immune recognition of DAMPs was followed by upregulation of muscle tissue repair genes (Neubauer et al. 2014). In another recent study, mice completed a fatiguing treadmill exercise, which increased blood neutrophil (and monocyte and lymphocyte) numbers by 12 h post exercise, and skeletal muscle showed increased expression of adhesion molecules (Nunes‐Silva et al. 2014). Increased numbers of rolling, adherent, and transmigrating neutrophils interacted with endothelial cells, and with exercise, they were recruited into the quadriceps muscle, as a result of the increased levels of adhesion molecules (Nunes‐Silva et al. 2014).

Considering these studies in conjunction with results of the present study, it is possible that as numbers of neutrophils and levels of their TLRs increase, that neutrophils act as a sink for uptake of cf‐mDNA in the blood, absorbing it and carrying it to muscle tissue. There, cf‐mDNA, known to activate neutrophils, would help promote exercise‐induced neutrophil migration, inflammation, cellular damage, and/or tissue repair. The production of IL‐6 by exercising muscle cells and its inflammatory/anti‐inflammatory properties are well documented (Pedersen and Febbraio 2012). This provides a marker of damage/inflammation/repair in our exercise trials that is consistent with other studies that have observed increased plasma IL‐6 with moderate intensity exercise (Cox et al. 2007).

Alternatively, cf‐mDNA has recently been shown to increase neutrophil adherence to endothelial cells and general permeability and endocytosis in endothelial cells in a neutrophil‐independent and neutrophil‐dependent manner (Sun et al. 2013). So, it is possible that uptake of cf‐mDNA by endothelial cells occurs with or without neutrophils.

Additionally, many substances, including IL‐6 (reviewed in [Pedersen and Febbraio 2012]), are cleared from the blood via the liver. Exercise, however, diverts blood flow away from the viscera and toward the skin and muscles, and although this is accompanied by increased cardiac output, increased blood flow to the liver, alone, probably cannot explain the reduction in cf‐mDNA observed (Shephard and Johnson 2015).

Finally, DNAse I activity might be involved in the clearance of cf‐mDNA. Beiter et al. (2014) showed that in response to exhaustive short‐term treadmill exercise, not only was there neutrophil extrusion of NETs and increased plasma cf‐nDNA but also a concurrent increase in plasma DNAse I activity immediately after exercise, proposed to clear NETs (Beiter et al. 2014). Thus, it is possible that the declines in cf‐mDNA that we detected during the prolonged exercise trial correspond to increased levels of DNAse I.

In vitro, neutrophils have been shown to extrude cf‐mDNA alone, or cf‐mDNA and cf‐nDNA in NETs, depending on the inducing agent (Yousefi et al. 2009; Keshari et al. 2012). However, whether cf‐mDNA post exercise may be derived from NETs with certain exercise protocols or whether NETs can trap circulating cf‐mDNA is unknown. Interestingly, cf‐mDNA induced by trauma was recently shown to activate NET formation in neutrophils via TLR9 (Itagaki et al. 2015). Thus, it is possible that during exercise‐induced neutrophil responses, NETs are activated by, release, or trap cf‐mDNA.

Response to prolonged moderate exercise does not produce as much muscle damage as strenuous weight lifting exercise that isolates eccentric (lengthening) muscle contractions (Clarkson and Hubal 2002; Howatson and van Someren 2008). We have also conducted a pilot experiment using an exercise damage protocol with two adult males who had resistance exercise training experience. After 10 sets/10 repetitions of eccentric bench press and eccentric leg press using 60% of an eccentric one‐repetition maximum load or until failure, declines in cf‐mDNA were also revealed (data not shown). One of these subjects reported feeling ill with elevated body temperature post exercise.

While generally, cf‐nDNA has been localized to soluble plasma fractions, cf‐mDNA was shown to be largely associated with particulate microvesicles in plasma pelleted 30 min at 10,000 g (Helmig et al. 2015). Our plasma samples for cf‐mDNA isolation were centrifuged 5 min at 800 g and 5–7 min at 2600 g (to remove platelets harboring mitochondria), so it is likely that microvesicle‐associated cf‐mDNA was retained in the samples. We cannot explain a slight variable increase in cf‐mDNA in the control trial, and effects of catheter placement or slight movement of the subjects cannot be ruled out.

Interestingly, there are reports of reduced plasma cf‐mDNA in some cancer studies, for example, (Kohler et al. 2009), and reductions in plasma cf‐DNA in established rheumatoid arthritis (RA) patients, for example, (Dunaeva et al. 2015), which ruled out DNAse I as a mechanism of cf‐DNA decline. This study postulated a role for methotrexate drug treatment (reduced nucleotide metabolism and DNA synthesis), increased metabolism and ROS production by blood cells, increased oxidative enzyme activity (or decreased antioxidants), and the presence of novel cf‐DNA forms resulting from RA‐specific influences on apoptosis, cell shedding, or nucleosomes, in the reduction in cf‐DNA observed (Dunaeva et al. 2015). Some of these mechanisms could also potentially play a role in cf‐mDNA reduction during prolonged exercise in healthy subjects.

While we observed a decline in cf‐mDNA with prolonged moderate aerobic treadmill exercise in seven subjects, it is notable that an eighth treadmill subject did not show this response to the aerobic exercise (data not shown). This subject clearly had increased levels of cf‐mDNA (241% baseline, with 18 min of exercise), which declined, but remained elevated relative to baseline throughout the trial. Since we suspected that this subject was responding differently from the other subjects, we subjected his cf‐mDNA data to two outlier tests, Grubbs and Fence method, which both failed; therefore, we excluded this eighth subject from the combined results. Notably, in contrast to all of the other subjects that had increased levels of plasma IL‐6 with 90 min of exercise, this subject had reduced levels of IL‐6 with exercise (e.g., 5% and 9% baseline at +54 and +90, respectively). It is possible that this subject was deficient in cf‐mDNA clearance with exercise and that this relates to the inability to subsequently induce an IL‐6 response in exercising muscles.

Of note, is that while several studies have seen increased cf‐DNA with different exercise protocols, most do not specifically examine cf‐mDNA and most examine levels post, but not during exercise (Fatouros et al. 2010; Beiter et al. 2011, 2014). Studies that addressed cf‐mDNA reported no change in cf‐mDNA levels after exhaustive short‐term treadmill exercise (Beiter et al. 2011; Helmig et al. 2015). The largest decline in cf‐mDNA observed in our treadmill exercise experiment (47.5% baseline) was at +54 during the exercise trial, with levels still reduced at the end of the trial (61% baseline) at +90 (immediately post), but regained by 40 min of recovery. It is possible that during aerobic exercise protocols, significant decline in cf‐mDNA occurs predominantly during prolonged protocols.

Several studies suggest that exercise can reduce incidence of disease by suppressing systemic inflammation (Gleeson et al. 2011; Lancaster and Febbraio 2014). cf‐mDNA is known to be an activator of systemic inflammatory processes. While we acknowledge that variables in measurement of cf‐mDNA that have perplexed and intrigued the field can influence results, our study suggests that transient clearance of cf‐mDNA can occur during the course of certain exercise protocols. We propose that such transient clearance events involving cf‐mDNA, repeated regularly, may provide health benefits by reducing systemic inflammation.

## Conflict of Interest

None declared.
